# Dual-tasking and gait in people with Mild Cognitive Impairment. The effect of working memory

**DOI:** 10.1186/1471-2318-9-41

**Published:** 2009-09-01

**Authors:** Manuel Montero-Odasso, Howard Bergman, Natalie A Phillips, Chek H Wong, Nadia Sourial, Howard Chertkow

**Affiliations:** 1 Department of Medicine, Division of Geriatric Medicine, Parkwood Hospital, The University of Western Ontario, London, ON Canada; 2 Lawson Research Institute, London, ON, Canada; 3 Division of Geriatric Medicine, Department of Medicine, McGill University and Jewish General Hospital, Montréal, QC Canada; 4 Department of Psychology, Concordia University, Montréal QC, Canada; 5 Department of Geriatric Medicine, Singapore General Hospital, Singapore; 6 Department of Neurology and Neurosurgery, McGill University, Montréal, QC Canada; 7 Solidage: McGill University/University of Montréal Research Group, Montréal, QC Canada

## Abstract

**Background:**

Cognition and mobility in older adults are closely associated and they decline together with aging. Studies evaluating associations between cognitive factors and gait performance in people with Mild Cognitive Impairment (MCI) are scarce. In this study, our aim was to determine whether specific cognitive factors have a more identifiable effect on gait velocity during dual-tasking in people with MCI.

**Methods:**

Fifty-five participants, mean age 77.7 (SD = 5.9), 45% women, with MCI were evaluated for global cognition, working memory, executive function, and attention. Gait Velocity (GV) was measured under a single-task condition (single GV) and under two dual-task conditions: 1) while counting backwards (counting GV), 2) while naming animals (verbal GV). Multivariable linear regression analysis was used to examine associations with an alpha-level of 0.05.

**Results:**

Participants experienced a reduction in GV while engaging in dual-task challenges (p < 0.005). Low executive function and working memory performances were associated with slow single GV (p = 0.038), slow counting GV (p = 0.017), and slow verbal GV (p = 0.031). After adjustments, working memory was the only cognitive factor which remained significantly associated with a slow GV.

**Conclusion:**

In older adults with MCI, low working memory performance was associated with slow GV. Dual-task conditions showed the strongest associations with gait slowing. Our findings suggest that cortical control of gait is associated with decline in working memory in people with MCI.

## Background

Cognitive problems in older adults range from mild impairment to severe dementia. The transitional stage between normal aging and dementia has been designated as Mild Cognitive Impairment (MCI) [1-3]. People with MCI have been found to have a 10 to 15 times higher risk of developing Alzheimer's disease (AD), although up to 40% will not develop dementia [4,5]. Prevalence of MCI is estimated at 19% among older adults, increasing to 29% in those over age 85 [6]. The prevalence of gait disorders also increases with age, with estimates of up to 20% in older people residing in the community [7]. Previously, age-associated slowing gait has been considered a benign consequence of aging; however, slow gait velocity has been recently associated with increased risk of falls, institutionalization, and mortality [8,9]. Interestingly, gait slowing and cognitive impairment usually coexist in the same individual and the interaction between cognitive impairment and motor changes in older adults has been established [10-12]. This interrelationship has been attributed to specific brain networks selectively affected by diseases that accompany, but are not necessarily caused by, ageing [13].

Gait is a complex learned task which has been considered almost automatic with limited involvement of cognitive control. However, recent studies have established the importance of cognitive control on gait in older adults [7,14], although the complexity of this interaction is not yet fully understood [15,16]. For example, the interdependence between gait and cognition in older people is manifested in the fact that slow gait performance is more prevalent in people with cognitive impairment and dementia [12,16-19]. Similarly, slow gait in healthy older adults has also been associated with higher risk to develop cognitive decline and dementia [20,21].

A sensitive way to detect these early interactions is to measure the effect that a cognitive load (e.g. simultaneous talking or counting while walking) has on gait. Since one seminal study demonstrated that the inability to maintain a conversation while walking ("stops walking while talking") is a marker for future falls in older adults [22], walking while performing a secondary task (dual-task paradigm) has become the classic way to assess the interaction between cognition and gait. In the past, it has been established that the effect of dual-tasking on gait velocity (dual- task decrement) is related to impairments in executive function and attention. For example, patients with Alzheimer's Disease and patients with Parkinson's Disease who have more impediments in executive function show a greater dual-task decrement [23-25]. This suggests that the cognitive reserve may play an important role while performing dual-tasks. On the other hand, a dual-task decrement is also seen in healthy older adults, but is much less pronounced.

Attempting to isolate the specific cognitive factors which impact mobility in people with Alzheimer's Disease, Parkinson's or neurological disease can be difficult because of the global nature of their cognitive impairment. A way to elucidate these associations is to target a population with early cognitive impairments. Since people with MCI do not meet the criteria of dementia and generally have limited cognitive deficits in one or more domains, they may be at the optimum stage to evaluate these interactions.

Studies evaluating associations between cognitive factors and gait performance in older people with MCI are limited. Improved characterizations of these associations are important to advance our understanding of the early interactions between gait and cognition in this population and may potentially assist in the detection of those individuals who are at higher risk of future mobility decline (e.g. falls) and cognitive decline (e.g. dementia). Therefore, the present study was designed to evaluate the effect of specific cognitive factors (executive function, memory, and attention), on gait velocity (GV) in people with MCI. We hypothesize that the greatest effect of the cognitive factors on gait velocity will be seen under dual-task conditions.

## Methods

### Participants

Sixty older adults with MCI enrolled in an ongoing longitudinal study at the Jewish General Hospital Memory Clinic, McGill University (Montréal, Canada) were contacted by phone and invited to participate. Inclusion criteria were: age 65 and older, having a diagnosis of MCI, and fluent in English. All the participants belonging to this cohort were diagnosed with MCI based on clinical criteria [4,26], which included the presence of subjective memory complaints from the patient and family, objective memory impairment, preserved general intellectual function (assessed clinically), absence of significant functional impairment, and absence of clinical dementia. Objective memory impairment was operationalized by the demonstration of memory impairment by neuropsychological testing on standardized memory tests, as suggested by a recent consensus [27] (specifically, scores on Logical Memory 2 of the Wechsler Memory Scale-Revised and/or the delayed recall score of the Rey Auditory Verbal Learning Test that were 1.5 standard deviations below age-adjusted norms). When participants were included in the present study, the diagnosis of MCI was corroborated based on review of their cognitive tests, absence of dementia according to Diagnostic and Statistical Manual of Mental Disorders, Fourth Edition (DSM-IV) criteria [28], and absence of functional impairment as indicated by normal scores on the Lawton Brody Scale [29]. Exclusion criteria included: any objective gait disorder due to Parkinson's disease, previous stroke, clinical osteoarthritis in lower limb joints, myopathy, or neuropathy as verified by a formal clinical examination. The presence of depressive symptoms, defined as a score ≥ 5/15 on the Geriatric Depression Scale [30], was also an exclusion criterion since depression may affect gait performance [31]. The Institutional Review Board at Jewish General Hospital and McGill University approved the study.

### Medical and cognitive assessments

Participants who provided informed consent had a comprehensive medical interview for comorbidities, medications, history of falls in the previous 12 months, and fear of falling. History of falls and fear of falling was self reported. Global cognitive status was assessed using the Mini Mental State Examination (MMSE; scored 0-30) [32], which was used for descriptive purposes but not in analyses. The Montreal Cognitive Assessment (MoCA; scored 0-30, with a higher score indicating better performance) was used to evaluate global cognition and, the subset of items measuring delayed recall of the five words (scored 0-5) was used to evaluate memory. The MoCA test is a validated tool used to assess global cognition and was originally created to assist in the diagnosis of MCI [33]. In brief, when considering MMSE and MoCA performance in the same individual a pattern of low MoCA score (< 26) with normal MMSE score (> 26) is associated with MCI [33]. Psychomotor speed was assessed with the Digit Symbol Test (scored 0-133, with higher scores indicating better performance) [34] which evaluates the speed with which participants copy arbitrary symbols that are paired with digits. Executive functioning was assessed using the Trail Making Test, forms A and B (TMT A & B; scored in seconds, with more time indicating a worse performance). The TMT is a well-established, timed, psychomotor test which has been widely used in clinical evaluations for the assessment of deficits in attention and executive cognitive functions and is administered in two parts [35]. For TMT form A, the participant is required to draw lines sequentially connecting numbered circles arranged randomly on a page as quickly as possible. TMT form B is a more demanding task as it requires the participant to connect circles containing numbers and letters in an alternating sequence (e.g. 1 - A - 2 - B - 3 - etc.) [36]. TMT form B is considered to reflect aspects of executive function due to the mental flexibility required to alternate between the two stimuli categories. TMT B-A. was used in order to isolate the executive functioning component since it minimizes visuoperceptual and working memory demands, providing a relatively pure indicator of executive control abilities [37]. Working memory was evaluated using the Letter Number Sequencing test (LNS; scored from 0 to 21, with a higher score indicating a better performance) which examines the ability to retain and process a sequence of letters and numbers. The participant was read a combination of numbers and letters and asked to repeat them, saying the number first in ascending order and then the letters in alphabetical order [38]. Trained research assistants administered all the cognitive tests. After completing the cognitive evaluations, participants underwent the gait assessment conducted by a geriatrician who was blinded to the results of the cognitive tests.

### Gait Assessment

Gait was assessed using the gait velocity test (GV) which is a highly reliable and reproducible measure of mobility in older individuals with good functionality [39]. The walking trials were performed in a well-lit, 10-meter long hallway. Gait velocity was measured as the time taken to walk the middle 6 meters of 8 meters and was timed in centiseconds using a stopwatch (PTFitness Professional chronometer). Starting and ending limits were marked on the floor with tapelines. The first and last meters, considered warm-up and the deceleration phases, respectively, were not included in the calculation as per our gait assessment protocol described elsewhere [9,40].

After giving standardized instructions and a visual demonstration, participants were instructed to walk at a comfortable pace at their usual speed. Following one un-timed practice trial, each participant performed three different timed walking trials. One trial measured the gait velocity at a self-selected pace: this was the single-task condition (single GV). Two trials measured gait velocity while performing a verbal or counting task (verbal GV and Counting GV, respectively). During verbal fluency dual-task condition (verbal GV), we measured the gait velocity while participants were naming animals aloud; during the arithmetic dual-task condition (counting GV) we measured gait velocity while participants were counting backward aloud from one hundred by ones. These two different dual-task conditions were selected based on previous research which demonstrated that counting backwards by one is an almost automatic task which depends more on working memory and attention [41], while naming animals out loud is more related to verbal fluency, which relies on semantic memory [42]. To reduce learning effects, participants were given only one untimed practice trial as required on both the single and dual-task conditions to familiarize them with the procedure. The order of the trials was counterbalanced and while performing dual-tasking trials there was no instruction to prioritize gait or cognitive task. Allowing both gait and cognitive tasks to vary has previously been shown to provide a better representation of daily living activities [25,43].

### Statistical Analysis

Power and sample size calculations were conducted using the PROC POWER procedure, MULTREG option for multiple linear regression. A sample size of 60 subjects was estimated to provide > 70% to detect a moderate to large effect of the predictors of interest. Baseline characteristics were descriptively summarized using either means and standard deviations or frequencies and percentages, as appropriate. Cross-sectional associations between cognitive function and GVs were tested using linear regression models, both unadjusted and adjusted for age, sex, and history of falls. Statistical significance was set at 0.05 and analyses were conducted using SAS 9.1 (SAS Institute Inc., Cary, NC).

## Results

**Figure 1:**
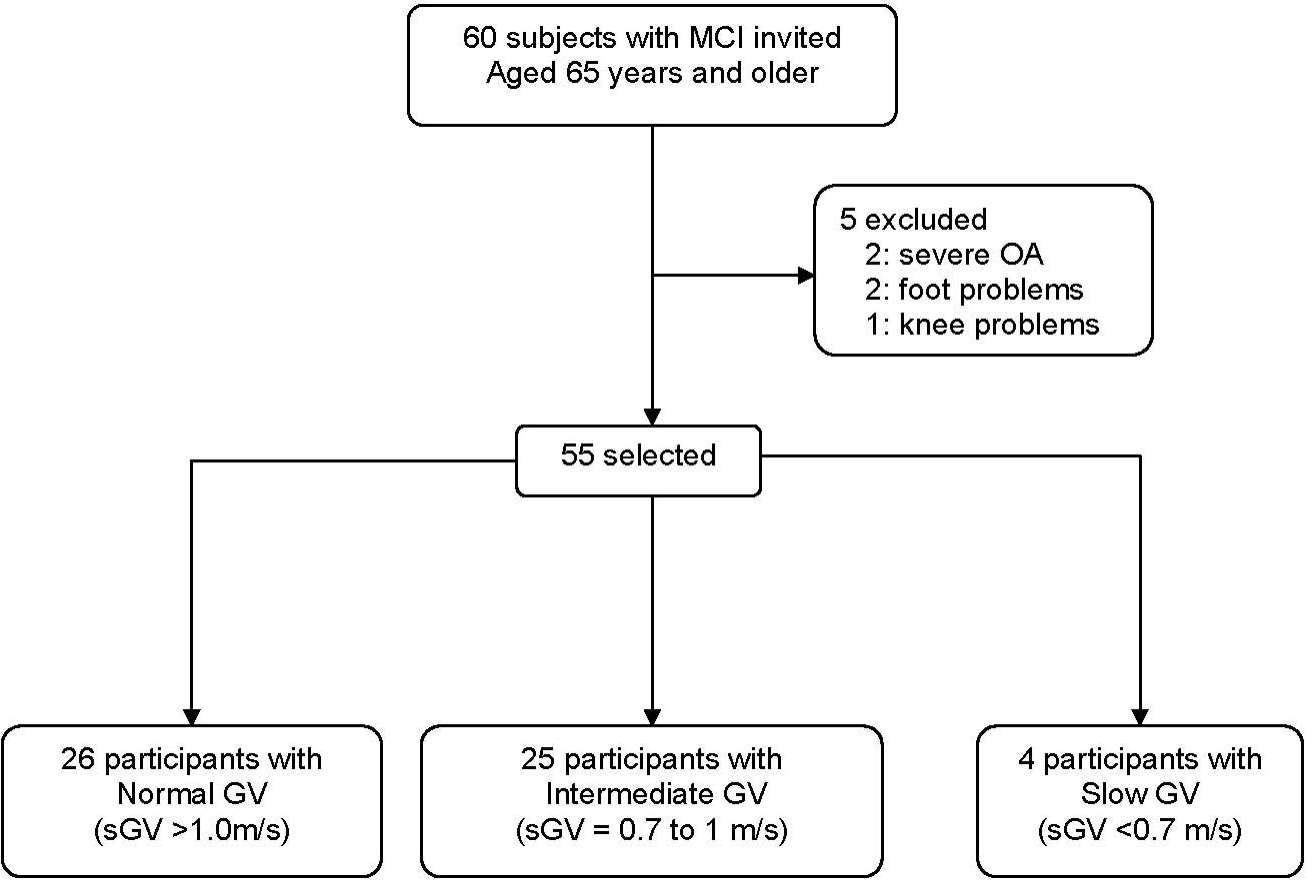
**Selection of study participants**. **Note**: MCI: mild cognitive impairment; GV: gait velocity; sGV: single task gait velocity; OA: osteoarthritis.

Sixty subjects with MCI were contacted and all agreed to participate in the study. Five individuals were excluded due to comorbidities that affected the gait performance. The assembly of the cohort is schematized in Figure 1 and divided in groups according to their gait velocity (GV) for descriptive purposes, based on cut-offs determined in previous studies[9,39,44]. Of study participants, 47.25% had a normal GV (> 1 m/s), 45.45% an intermediate GV (between 1 and 0.7 m/s) and 7.27% a slow GV (< 0.7 m/s)

### Participant Characteristics

Demographic characteristics of the participants, as well as their performance on cognitive and gait assessments, are summarized in Table 1. Participants' mean scores on global cognition tests were consistent with the diagnosis of MCI since a pattern of low MoCA score (< 26) with normal MMSE score (> 26) was found in our sample [33]. Mean performance in TMT A, Digit symbol and LNS was within normal ranges while the mean performance in TMT B was below the normative data for older subjects [45].

**Table 1:** Characteristics of the participants (n = 55).

Characteristics	Mean (SD); n (%)	Range:
Age in years	77.7 (5.89)	66-90
Women	25 (45.5%)	N/A
At least 1 fall in last 12 months	14 (25%)	N/A
BMI	25.8 (4.4)	14-42
Years of education	12.1 (3.4)	(8-20)
Cognition		
MMSE Score (0-30)	26.8 (2.1)	21-30
MoCA score	22.4 (3.2)	15-28
Letter Number Sequencing (LNS)	7.6 (2.4)	3-13
TMT A (seconds)	55.8 (20.9)	23-102
TMT B (seconds)	178.8 (108.6)	55-539
Digit Symbol Coding	41 (14.4)	19-71
Gait Velocity (m/s)		
Single Gait Velocity (sGV)	0.87 (0.2)	0.44-1.50
Verbal Gait Velocity (vGV)	0.65 (0.2)	0.32-1.00
Counting Gait Velocity (cGV)	0.63 (0.2)	0.34-1.06

**Note**: SD = Standard deviation; N/A = not applicable; MMSE = Mini-Mental State Examination; MoCA = Montreal Cognitive Assessment; TMT = trail making test.

### Gait Assessments

Mean single GV was 0.87 m/s (SD = 0.2) and there were no significant differences in gait velocity regarding age and sex.

### Effect of Dual-Tasks on Gait

**Figure 2:**
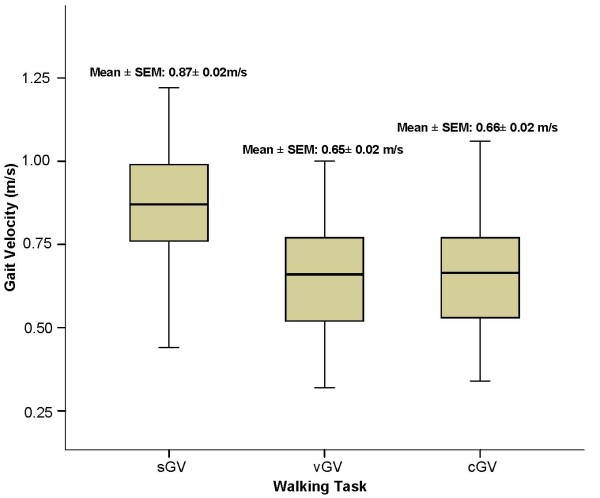
**Mean gait velocity under single (sGV) and dual tasks (vGV, cGV)**. **Note**: sGV: single task gait velocity; vGV: verbal gait velocity; cGV: counting gait velocity.

Participants experienced a significant decrease in gait velocity while engaging dual-task conditions when compared with single GV (p < 0.0001, Figure 2). A significant correlation between GV under both types of dual-task (verbal GV = 0.65 m/s, SD = 0.2, and counting GV = 0.63 m/s, SD = 0.2, r = 0.89) was found. The associations between GVs under dual-tasks and the cognitive functions explored on the multivariable analysis were analogous, as presented in Additional file 1.

### Associations between Cognitive Factors and Gait Velocity performance

Associations were explored unadjusted and adjusted to specifically look at the effect of confounders. A significant association was found between the Trail Making Test B and the three GVs explored (single GV, p = 0.038; verbal GV, p = 0.031; and counting GV, p = 0.017, respectively) in the unadjusted analysis. A negative correlation between executive function (TMT B) and the three GV (r = -0.47, p-value = 0.038) was found with a parameter estimate of -0.0004, which means that for every 100-second increase in the TMT B performance, GV went down by 4 cm/s, which is a clinically relevant change on GV [46]. The range of the TMT B was between 55 and 539 seconds in our participants. The upper quintile has a mean of 60 seconds while the lowest quintile a mean of 400 seconds. Therefore, the differences between lower and upper quintile in the TMT B performance is in the range of 320 seconds which represent a change on GV of 13 cm/s.

After adjustments, TMT B remained significantly associated with counting GVs (p = 0.04, see Additional file 1). When we corrected TMT for the attentional component using the TMT B-A, a significant association with low counting GV was found (p = 0.038). Low performance on the digit symbol test was marginally associated with dual-tasks GVs in the unadjusted and adjusted analyses [see Additional file 1]. LNS test performance was the only test consistently associated with single and dual-tasking decrement on GVs in the unadjusted and adjusted analyses (p < 0.05, see Additional file 1).

Overall, dual-tasking decrements on GV were associated with lower performance in cognitive factors when compared with single tasking gait performance [see Additional file 1]. No associations were found between global cognitive scores (MMSE and MoCA), TMT A, and the delayed recall component of the MoCA test and GV under single or dual-tasks. The effect of grouping participants in amnestic and non-amnestic MCI was explored and no significant differences were found, although the study was not powered for this analysis.

## Discussion

This study established the effect of specific cognitive factors on gait performance in older adults with MCI. Specifically, dual-tasking decrement on gait velocity was significantly associated with low performance in executive function and working memory. The decrement on gait while dual-tasking was significant when compared with single-tasking gait performance.

The significant decline on GV while engaging in dual-tasking found in our participants is in agreement with previous research which shows that gait performance in older persons may be increasingly dependent on cognitive control when compared with younger adults [47-49]. We found a significant correlation between verbal GV and counting GV, suggesting that the effect of dual-task on GV was independent of the type of task demand selected for this study. Participants with low performance in executive functioning experienced a greater slowing in their GV, similar to what has been already described in cognitively intact older adults [15,50]. When we adjusted for the history of previous falls, this association remains only with counting GV. One possible explanation, suggested by previous studies, is that the history of previous falls can be a marker of poor executive function[9,51,52]. This is also supported by the significant negative correlation found between executive function and GV in our study.

An interesting finding was the significant association between working memory and slowing gait velocity. Previously, the attention/executive factor emerged consistently as the most robust predictor of slowing of gait[14,49,50,53-57]. Here, working memory was the only factor which remained significantly associated with slowing gait after adjustments. This may suggest that in the MCI population, working memory is one of the first cognitive factors which may evidence deterioration under dual-tasking. Working memory is one component of executive control found to be necessary for sequential ordering movement [38,58,59]. The association between executive function, specifically working memory, and slowing gait may suggest that both functions are controlled by the same specific areas of the cortex. We hypothesized that occupying these areas with concurrent cognitive processing may result in a brain resource limitation that affects gait in people with MCI. Specifically, dual-tasking cost has been traditionally related to attentional function and to the prefrontal cortical regions. These brain regions are crucially involved in the mediation of the division of attention and executive function. Functional neuroimaging studies showed correlations between dual-task performance with increase activity in prefrontal areas, cingulate, parietal and premotor areas [60].

**Figure 3:**
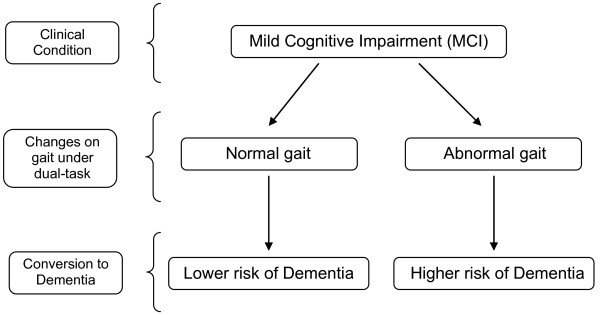
**Proposal that slowing gait velocity could be an early manifestation of progression to dementia in people with MCI**.

Additionally, previous research has found that working memory is one of the first functions compromised in Alzheimer's Disease [61,62], and a recent study showed that working memory impairment can be a marker of progression to dementia in people with MCI [63]. Within this framework, our data suggest that dual-tasking decrement on gait is an early phenomenon in people with MCI occurring concurrently with the cognitive decline. We hypothesize that dual-task decrements may help on the detection of those individuals at higher risk of progression to dementia (figure 3). A further longitudinal study is needed to demonstrate this hypothesis.

This study has the strength of including a well-defined population meeting strict criteria for MCI, and the use of established and validated measures of cognition and gait. However, limitations include the use of a cross-sectional rather than longitudinal design, which limited our ability to assess temporality of potential cause and effects, and the lack of a control group. Gait velocity was assessed using a stopwatch and without using an electronic walkway; therefore, other quantitative gait variables beyond velocity were not available for analysis. As falls history was based on self-report, this increased the likelihood that the number of falls was underreported. Despite these limitations, we found an effect of cognitive dysfunction, particularly working memory, on gait performance in this population.

## Conclusion

In summary, we found significant associations between executive function, working memory and gait slowing in older people with MCI, which were better detected under dual-tasking conditions. Poor working memory was strongly associated with dual-task decrement on gait velocity which may express an early phenomenon in people with MCI. These associations hold clinical and anatomical plausibility. Our results support that quantitative examination of gait under dual-tasking is a complementary way to evaluate brain function during the preclinical onset of dementia. Since gait may be more easily and quantifiably measured than psychometrics, our findings might contribute to establish the value of quantitative gait assessment as a complementary measure to neuropsychological assessment in older adults with MCI.

## Competing interests

The authors declare that they have no competing interests.

## Authors' contributions

Study concept and design (MMO and HB), acquisition of subjects and data (MMO, NP, CW), data analysis (MMO, NS, CW), preparation of the manuscript (MMO), and critical review of the manuscript (MMO, HB, CW, NS, NP, HC). All authors read and approved the final manuscript.

## Pre-publication history

The pre-publication history for this paper can be accessed here:



## Supplementary Material

Additional file 1 **Linear regression analysis of the associations of cognitive tests and GV with single and dual-task conditions**. Data provided represents results of a linear regression analysis of the associations of cognitive tests and GV with single and dual-task conditions. Click here for file
