# Supplementary material for: Dual-tasking and gait in people with Mild Cognitive Impairment. The effect of working memory
Source: BMC Geriatr. 2009 Sep 1;9:41. doi: 10.1186/1471-2318-9-41 (PMC2748075; doi:10.1186/1471-2318-9-41)
Supplement: Additional file 1 — Linear regression analysis of the associations of cognitive tests and GV with single and dual-task conditions. Data provided represents results of a linear regression analysis of the associations of cognitive tests and GV with single and dual-task conditions. [file 1471-2318-9-41-S1.doc]

***Additional file 1.*** *Linear regression analysis of the associations of cognitive tests and GV with single and dual-task conditions.*

|  |  | **Single Gait Velocity** | | **Verbal Gait Velocity** | | **Count Gait Velocity** | |
| --- | --- | --- | --- | --- | --- | --- | --- |
| **Test** |  | **Estimate ± SE** | **p-Value** | **Estimate ± SE** | **p-Value** | **Estimate ± SE** | **p-Value** |
| Delayed MoCA  (memory) | Unadjusted | -0.006 ± 0.014 | 0.653 | 0.0087 ± 0.015 | 0.547 | -0.0014 ± 0.015 | 0.928 |
|  | Age, Gender | -0.010 ± 0.015 | 0.502 | 0.0068 ± 0.016 | 0.668 | -0.0008 ± 0.017 | 0.963 |
|  | Falls | -0.014 ± 0.015 | 0.367 | 0.0052 ± 0.017 | 0.748 | -0.0048 ± 0.017 | 0.774 |
| TMT A  (attention) | Unadjusted | -0.001 ± 0.001 | 0.349 | -0.002 ± 0.001 | 0.150 | -0.002 ± 0.001 | 0.094 |
|  | Age, Gender | -0.001 ± 0.001 | 0.453 | -0.002 ± 0.001 | 0.195 | -0.002 ± 0.001 | 0.060 |
|  | Falls | -0.001 ± 0.001 | 0.618 | -0.002 ± 0.001 | 0.244 | -0.002 ± 0.001 | 0.103 |
| TMT B  (executive function) | Unadjusted | -0.0004 ± 0.002 | 0.038* | -0.0004 ± 0.002 | 0.031* | -0.0005 ± 0.0002 | 0.017* |
|  | Age, Gender | -0.000 ± 0.0002 | 0.057 | -0.0004 ± 0.0002 | 0.045* | -0.0005 ± 0.0002 | 0.017* |
|  | Falls | -0.000 ± 0.0002 | 0.125 | -0.0004 ± 0.0002 | 0.067 | -0.0005 ± 0.0002 | 0.040* |
| TMT B – A  (pure executive function) | Unadjusted | -0.000 ± 0.000 | 0.060 | -0.000 ± 0.000 | 0.061 | -0.000 ± 0.000 | 0.038* |
|  | Age, Gender | -0.000 ± 0.000 | 0.080 | -0.000 ± 0.000 | 0.079 | -0.000 ± 0.000 | 0.041* |
|  | Falls | -0.000 ± 0.000 | 0.188 | -0.000 ± 0.000 | 0.126 | -0.000 ± 0.000 | 0.098 |
| Digit Symbol  (psychomotor speed) | Unadjusted | 0.002 ± 0.002 | 0.148 | 0.0033 ± 0.0016 | 0.042* | 0.0032 ± 0.002 | 0.053 |
|  | Age, Gender | 0.002 ± 0.002 | 0.188 | 0.0036 ± 0.002 | 0.049* | 0.0039 ± 0.002 | 0.034* |
|  | Falls | 0.002 ± 0.002 | 0.317 | 0.0035 ± 0.002 | 0.065 | 0.0036 ± 0.002 | 0.062 |
| LNS  (working memory) | Unadjusted | 0.024 ± 0.0080 | 0.004* | 0.0223 ± 0.0083 | 0.010* | 0.0270 ± 0.008 | 0.002* |
|  | Age, Gender | 0.024 ± 0.008 | 0.005* | 0.0225 ± 0.0087 | 0.013* | 0.0284 ± 0.009 | 0.002* |
|  | Falls | 0.023 ± 0.008 | 0.009* | 0.0220 ± 0.0089 | 0.027* | 0.0274 ± 0.009 | 0.003* |

**Note**: *: Statistically significant (p < 0.05); SE: standard error; TMT: trial making test; LNS: letter number sequencing; delayed MoCA: delay recall of the Montreal Cognitive Assessment; Previous falls: at least one fall in last 12 months.
